# Human Physiology

**Published:** 1833

**Authors:** 


### Art. IV.—Human Physiology.

*Human Physiology Illustrated by Numerous Engravings.* By Robert Dunglison, M. D. Professor of Physiology, Pathology, &c. in the University of Virginia. Philadelphia: Carey & Lea. 1832. 2 vols. octavo, pp. 1046.

BY A CORRESPONDENT.

Of the numerous subjects which have engaged the attention of the votaries of science, there is none more universally attractive than the science of Physiology. Whether we consider the personal interest we feel in the subject, the delicacy of Nature's workmanship, the numerous proofs of art and design, insensibly, but unavoidably leading the mind to the Great First Cause, that made and sustains the Universe, we are led on with increasing interest to discover new beauties as the subject opens upon us. Nor is the interest lessened by the obscurity of the *Final Causes* of many of the most important functions of the Animal œconomy. On the contrary, the impenetrable veil which Nature has here thrown over her operations, has served to swell the number, and increase the ardor of those who are determined to push their investigations to the utmost limit of human penetration.

During the last half century, the science of Physiology has been investigated, both in this country and in Europe, with great success, its boundaries have been widely extended, and it has been disburthened of many vain speculations which served only to obstruct the progress of the student, by rendering the object of his pursuit, intricate and repulsive. While numbers of our countrymen have honorably engaged in forwarding the different branches of this important department of Medical science, it must be gratifying to the American student, that one of our associates in scientific research, with talents and learning fully competent to the task, has at length undertaken to embrace the whole subject, and place it before him with all the improvements which modern research has given it.

The first chapter of the work is devoted to the difference between organic and inorganic bodies, and between animals and vegetables. As the first is less immediately connected with our subject, we shall pass to the second—the discrimination of which, although at first view, apparently so obvious, is, when we come to compare the lower forms of animal with the higher forms of vegetable life, attended with very considerable difficulty, we shall therefore briefly allude to them.

1. Animals and vegetables differ in their *composition.* To the carbon, oxygen and hydrogen, in combination with earthty and metallic salts, which make up the substance of vegetables, azote is added in animals. When this latter substance is found as a component part of vegetables, it is generally confined to a part, and not distributed throughout the whole. In consequence of this difference of composition, the two substances are readily distinguished. The smell of burnt sponge, coral, or other zoophytic animal is so peculiar, that it can scarcely be mistaken for any vegetable substance.

2. They differ in their *texture.* Vegetables have a greater proportion of solids, animals of fluids, vegetables have but one tissue, the cellular—animals, in addition, have the muscular and the nervous.

3. Vegetables have neither *sensation* nor *voluntary motion*, the spontaneous movements which we observe in certain plants depending entirely upon organic irritability without consciousness.

4. They differ in their mode of *nutrition.* The absorbing vessels of vegetables open upon their surface—in animals the food after digestion is absorbed from the surface of the alimentary canal.

5. They differ in their mode of reproduction. It is not necessary, however, to point out the contrast between their sexual organs, their mode of fecundation, and the circumstances attending the birth of the new individual. These differences in function are not, however, as striking characteristics as the first view they appear to be. The zoophytite is as destitute of sensibility and locomotion as the vegetable, and accordingly no traces of nervous or muscular structure have been detected in them; and on the other hand, the spontaneous motions of the vegetable have been considered by some as the rudiments of sensibility and locomotibility.

In speaking of the *Physical Properties of the Tissues*, Dr. D. alludes to a very important property, to which much attention has been directed of late—that of *imbibition*. If a liquid be placed in contact with any animal tissue, after a time, it will be found to have penetrated its structure as it would enter the cells of a sponge. The length of time occupied in this imbibition will depend upon the nature of the fluid, and kind of tissue. The serous membranes and small vessels absorb with great promptitude—the epidermis on the contrary, resists imbibition for a considerable time. Transudation does not differ in principle from imbibition; it is technically applied, however, to the passage of fluids from within outwards.

Within a few years, some singular facts have been observed regarding the imbibition of fluids, and gases, by filling a membranous expansion, as a piece of intestine, with milk and immersing it in water, Dutrochet observed that the milk left the intestine, while the water entered it. Dutrochet hence inferred that whenever an organized cavity containing a fluid is immersed in a fluid less dense, it has a tendency to expel the denser and absorb the rarer fluid. This, Dutrochet termed, endosmose or inward impulsion, and he conceived it to be the result of vital action. Subsequent experiments have shown that a reverse action may take place. The transmission, when the internal fluid is rare, he called exosmose or outward impulsion. The discovery of this curious principle led the ardent imagination of Dutrochet to form many curious speculations. Among others, he supposed the energy of action both in exosmose and ednosmose, to be in proportion to their density and their chemical nature, and that it depended upon an electrical influence.

Dr. Mitchell, however, has shown by numerous experiments, that imbibition and transudation are dependent upon the penetrativeness of the fluids, and the penetrability of the membrane; and, consequently, are entirely independent of a difference in specific gravity, or electrical affinity. Thus, if two fluids of different degress of penetrativeness be placed on opposite sides of an animal membrane, they will in time present the greatest accumulation on the side of the less penetrative, but will finally thoroughly and uniformly mix upon both sides; and, at length, if any pressure exist upon either side, yield to that and pass to the other side.

The same principle holds good in relation to gases. A bladder filled with hydrogen and placed in contact with atmospheric air, will permit the hydrogen to escape, and absorb common air. Dr. M. found that some gases were transmitted much more readily than others. The more fresh the membrane, the more speedy and extensive was the effect, and in living animals it took place very rapidly. The tendency to pervade the membrane, also was so powerful, that it took place in opposition to a pressure equal to twice the weight of the atmosphere.

The strong tendency of elastic fluids to mix freely and perfectly, when placed in contact, has long been known to chemists and natural philosophers, but the discovery of the disposition, both of elastic and inelastic fluids, to unite through the medium of animal membrane, has a much more important bearing upon the subject of Physiology, since, in all probability, it presides over the functions both of respiration and absorption.

The principle is a very interesting and perhaps an inexplicable one. It may possibly be owing to a galvanic influence, but it cannot be attributed to vital action, for it continues long after every trace of vitability has ceased, and indeed until the animal texture is destroyed by chemical changes.

In treating of the functions, Dr. D. adopts the classification of Magendie, dividing them into 1. *The Functions of Relation*, including the *sensations*, *voluntary motions*, and *expressions;* 2. The Functions of Nutrition; and, 3. The Functions of Generation.

In studying each of these functions, he describes the organ or apparatus, concerned in its production, so far as is necessary in a physiological point of view; and where external agents are concerned, as light in vision, sound in hearing, he gives the general principles of physic which are necessary to a full elucidation of their action.

As the principal agent in the *functions of relation*, the nervous system comes first under the consideration of the author. In considering this branch of the subject, it is impossible for the most indifferent observer not to be struck with the beautiful and varied contrivances to which nature has resorted, for the purpose of protecting the brain and spinal marrow from injury. The brain is defended from the injuries to which, in consequence of the exposed situation of the head, it is continually liable, by a bony case composed of two laminæ, which are separated by an inelastic cellulated texture, that prevents a shock upon the surface from being communicated to the brain. The principal source of danger to the spinal marrow, arises from the extent of motion which is necessary in the bony case which protects it, and from the shock produced by violent motions of the body. Against these dangers it is protected by the numerous joints of the spine, admitting of great latitude of motion, without much flexure at any one point, by the elasticity of the intervertebral substance, enabling the spine to yield to any violence, and gradually to recover its position without injury, by the great extent and firmness of its articulations preventing dislocation. There is also another protection, which was first pointed out by Majendie. The spinal cord is considerably less than the canal which contains it, and the intermediate space is filled with a serous fluid, which forms a great protection against the dangers to which it would be liable from sudden and violent flexure of the spine. To this fluid Majendie has given the name of cepalo-spinal. The elasticity of the membrane which contains it, by keeping up a constant and uniform pressure, adds greatly to the security of the important viscus it is designed to protect.

After a very lucid description of the brain and nervous system, the author proceeds to the Physiology of this important and interesting part of the animal structure. The brain is the seat of the intellect or, rather according to the opinion of many distinguished physiologists of the present day, the organ.

Its functions, however, are so far beyond the reach of human investigation, that the physiologist has little to do but to describe its structure, and classify and enumerate the phenomena which evidently depend upon its organization. Upon the Physiology of the nervous system a degree of light and interest has been thrown by the discoveries of Mr. Charles Bell, which has rarely fallen to the lot of any one individual to give to any department of science.

The medulla oblongata is divided, by Mr. Bell, into three portions, *an anterior, middle, and posterior;* and it is affirmed by Sir Charles, that the anterior gives rise to the nerves of motion, the posterior to the nerves of sensibility, while the middle portion sends forth a set of nerves which are destined to preside over the important function of respiration, and over the muscles of expression which give to the human countenance so marked a superiority over the lower animals.

According to this arrangement, all the nerves of the body may be divided into two great classes—the *original, primitive or symmetrical,* and the *irregular or superadded.* The fifth pair of cerebral and all the spinal nerves, belong to this class. They are found in every class of animals, from the zoophyte to man, running in a direction perpendicular to the longitudinal line of the body, and destined to impart sensibility and muscular power. They arise also by two roots, the anterior of which is the nerve of muscular motion, and the posterior of sensibility.

The other class of nerves—the irregular or superadded, arise from a single root, are irregular in their distribution, and destitute of that symmetry which characterises the first class. As nerves of respiration and expression, they are of course more numerous and varied in the higher orders of animals.

This discovery of Mr. Bell has thrown great light upon the obscure question respecting the cause of the complicated and irregular distribution of the nerves, and irresistibly leads to the hope that many other problems, which have hitherto taxed the ingenuity of physiologists in vain, may yet receive a satisfactory solution.

The *great sympathetic* with its branches, forms the third great division of the nervous system. This nerve is considered by Bichat to be the great nervous centre of the involuntary functions, and is hence termed by him the *organic nervous system*, in contra-distinction to the *animal nervous system*, which presides over the functions of animal life.

In relation to this system of nerves, several very important questions present themselves for consideration. This nerve has generally been considered as the principal source of the nervous influence distributed to the viscera. Weber, however, a German anatomist of distinction, asserts that there is reason to believe, that this is not the sole visceral nerve, but that the *par vagum* may share in the same function. According to him, the great sympathetic is less developed while the *par vagum* is increased in the lower forms of animal life, until at length the latter, as is the case with some of the molluscæ, is the only visceral nerve. This view is perhaps supported by the fact, now very generally admitted, that the par vagum is the nerve principally concerned in digestion.

What is the use of the ganglia every where found in the course of the great sympathetic nerve? The author coincides with the opinion of Johnson and Bichat, that they serve to render the organs which derive their nerves from them, independent of the will. Independent of the fact, however, that the motions of these organs are involuntary, we see nothing to support this opinion, and if the opinion entertained by many physiologists, that this important branch of the nervous system is distinct and independent, connected with the cerebral system, but not originating from it, this view is destitute of a shadow of foundation. At best, therefore, the opinion can only be considered as hypothetical.

The facts respecting the sensibility of the nervous system, are possessed of a very high degree of interest. It has generally been supposed that the brain, the centre of perception, was possessed of very great sensibility. This, however, is not the fact, recent experiments having satisfactorily determined that no pain is produced by the application of any kind of irritation. The nerves possess a species of sensibility adapted to the function they are destined to perform, and are insensible to any other kind of irritation. The posterior roots of the spinal nerves convey general sensibility to the different parts of the body, and are susceptible of very acute pain, when wounded or lacerated, but there are other nerves which are entirely devoid of general sensibility.

"This has given occasion to a distinction of nerves into those of *general* and of *special* sensibility. The nerves, that must be considered as insensible or devoid of general sensibility, are the optic, olfactory, and auditory. Each of these has, however, a *special sensibility*, and although they may exhibit no pain when irritated, they are capable of being impressed by appropriate stimuli, as by light, in the case of the optic nerve; by odours, in that of the olfactory; and by sound, in that of the auditory. Yet we shall find, that each of these nerves of special sensibility requires the influence of a nerve of general sensibility, the fifth pair. Many other nerves appear also devoid of sensibility, as the third, fourth, and fifth pairs; the portio dura of the seventh; the ninth pair of encephalic nerves; and, as has been shown, all the anterior roots of the spinal nerves."

The sensibility also varies very much in parts of the body which are supplied by the nerves of general sensibility—each part being furnished with that kind of sensation which is necessary to guard it against the injuries to which, from its office or situation, it is most exposed. Thus the ligaments of the joints may be cut or burnt without conveying pain, but how acute is the pain when they are lacerated? It is well remarked, therefore, by the author, that before we determine any part to be insensible, we must have subjected it to every kind of irritation.

It is satisfactorily proved by experiments, that perception takes place in the brain. Different physiologists have sliced away the brain and found that sensation continued until the knife reached the corpora quadrigemina, and, again, by slicing away the spinal cord from below, it is ascertained that sensibility continues until we reach the medulla oblongata. It is in this part, therefore, that we must place the cerebral organ of the senses. To this rule, however, there is one exception.

"The experiments of Rolando and Flourens, which have been repeated by Magendie, show, however, that the sight is an exception; that it is lost by the removal of the hemispheres. If the right hemisphere be sliced away, the sight of the left eye is lost, and *vice versa;* one of the facts proving the decussation of the optic nerves. The experiments of these gentlemen show that vision, more than the other senses, requires a connexion with the organ of the intellectual faculties—the cerebral hemispheres; and this, as MAGENDIE has judiciously remarked, because vision rarely consists in a simple impression made by the light, but is connected with an intellectual process, by which we judge of the distance, size, shape, &c. of bodies."

With this solution we must confess we are not entirely satisfied. Distinct vision, it is true, rarely consists in a simple impression made by the light, but is connected with an intellectual process; but the same remark may, to a certain extent, be applied to others of the senses, especially to tact and hearing. It will not be denied, we think, that the perfect exercise of either of these senses, presupposes an intellectual process. If, therefore, a direct communication with the organ of intellect be necessary in the former case, it is equally so in the latter. The fact is that the mind associates so readily with the impressions made upon any of the senses, that a more direct communication appears to be entirely uncalled for.

Various speculations have been formed respecting the source of the nervous influence. The author after discountenancing the opinions of the older physiologists, appears inclined to favor the idea of Young and Philip, that the *nervous fluid* (by which we are to understand not a material existence, but simply the medium of communication,) is electroid or galvanoid in its nature.

"Dr. BOSTOCK, however, has remarked, that before the electric hypothesis can be considered proved, two points must be demonstrated; first, that *every* function of the nervous system may be performed by the substitution of electricity for the action of the nerves; and secondly, that *all* the nerves admit of this substitution. This is true, as concerns the belief in the *identity* of the nervous and electrical fluids; but we have, even now, evidence sufficient to show their similarity, and that we are justified in considering the nervous fluid as electroid or galvanoid in its nature, emanating from the brain by some action unknown to us, and distributed to the different parts of the system, to supply the expenditure which must be constantly going on."

In enumerating the *External Sensations*, Dr. Dunglison adopts the popular division reducing them all to "some modification" of these five, tact or touch, taste, smell, hearing, and vision. On this subject we shall extract the following interesting and important observations.

"All these have some properties in common. They are all situated at the surface of the body, so as to be capable of acting with due facility on external bodies. They all consist of two parts; the one *physical*, which modifies the action of the body, that causes the impression; the other *nervous or vital*, which receives the impression, and conveys it to the brain. In the eye and the ear, we have better exemplifications of this distinction than in the other senses. The physical portion of the eye is a true optical instrument, which modifies the light, before it impinges upon the retina. A similar modification is produced by the physical portion of the ear on the sonorous vibrations, before they reach the auditory nerve, whilst, in the other senses, the physical portion forms part of the common integument in which the nervous portion is situated, and cannot therefore be as easily distinguished.

Some of them, again, are symmetrical; that is, composed of two separate and similar halves, united by a median line, as the skin tongue, and nose. The others, the eye and the ear, are in pairs; and this, partly perhaps, to enable us to judge of the distances of external objects. We shall find, at least, that there are certain cases, in which both the organs are necessary for accurate appreciation.

Two of the senses,—vision and audition,—have, respectively, a nerve of special sensibility; and until of late years the smell has been believed to be similarly situated. In the present state of our knowledge, we cannot decide upon the precise nerve of taste, although it will be seen that a plausible opinion may be indulged on the subject. The general sense of touch is seated in the nerves of general sensibility. All, however, seem intimately connected with one of the nerves of general sensibility;—the fifth encephalic pair,—and, in the case of those senses, more particularly, which possess nerves of special sensibility, it is found, that they are under the presidency of this nerve of general sensibility; for if it be cut, the function is abolished, although the nerve of special sensibility may remain entire.

Constituting instruments by which the mind becomes acquainted with external bodies, it is manifestly of importance, that the senses should be influenced by volition. Most of them are so. The touch has the pliable upper extremity, admirably adapted for the purpose. The tongue is moveable in almost any direction. The eye can be turned towadrs objects, in almost all positions, by its own immediate muscles. The ear and the nose possess the least individual motion; but the last four, being seated in the head, are capable of being assisted by the muscles, adapted for its movement.

Lastly. It is a common observation, that the loss of one sense occasions greater vividness in the others. This is only true as regards the senses which administer chiefly to the intellect,—those of touch, audition, and vision, for example. Those of smell and taste may be destroyed, and yet the intellectual senses may be uuinfluenced in their action.

The cause of the superiority of the remaining intellectual senses, when one or more has been lost, is not owing to any superior organization in these senses, but is another example of the influence of education. The remaining senses are attentively exerted to compensate for the privation, and become surprisingly delicate.

We proceed to the consideration of the separate senses, beginning with that of *tact* or *touch*, because it is the most generally distributed, and may be regarded as that from which all the others are derived. They are all, indeed, modifications of the sense of touch. In the taste, the sapid body; in the smell, the odorous particle; in the hearing, the sonorous vibration; and in the sight, the particle of light, must impinge upon or *touch* the nervous part of the organ, before sensation can, in any of the cases, be effected."

We question very much the philosophy of this last remark. At least, we are able to discover none, or at least but a very remote analogy between the mode in which these different senses are affected. As to the eye, it is at best very problematical whether there be any material contact at all through the medium of rays proceeding from the illuminated body; and as to the ear, the impression is doubtless made, not by the vibratory motions of the air acting immediately upon the auditory nerve, but by their action upon the physical mechanism of the ear while the nerve is secondarily affected in a manner very different from the impression made by bodies presented to the sense of touch. Indeed the sense of touch, considered as a *sense* distinct from the general sensibility of the system, is a compound sense, requiring, in order to render it of any avail, the aid of the muscular sense. We were rather surprised that Dr. D., in enumerating the external sensations, should have entirely omitted this, which appears to us to be as strictly entitled to a place in the classification, as any of those which he has mentioned. It has been very clearly shown by Dr. Brown, in his "Philosophy of the Human Mind," that it is to this sense we owe our appreciation of the weight, size, roughness, or polish of bodies that are presented to the touch, and, what renders it of much more indispensable service to the animal œconomy, that, by means of it, we are enabled to preserve the equilibrium of our bodies. It is necessary, therefore, not only to locomotion, but to every change of the body from the recumbent position. Dr. D., however, appears to include the latter sense in the sense of touch. It (*the sense of touch*) does not exist in every animal, and is nothing more than tact joined to muscular contraction and directed by volition. So that in the exercise of tact, we may be regarded as *passive*; in *touch* as *active*. The two senses, however, are evidently not to be confounded, since we resort to the aid of the *muscular sense* under circumstances where the touch cannot possibly assist in the information we derive from it.

Dr. D. offers some very judicious observations upon the sense of touch, to which at present, we canscarcely make a reference. Some of his remarks, however, upon the supposed superiority of this sense, are so just and philosophical, that we shall make a short extract from them. After enumerating some of the errors to which this sense is liable, he makes the following judicious observations.

"It has been asserted, again, that the touch is the great corrector of the errors into which the other senses fall. But let us inquire whether, in this respect, it possesses any decided superiority over the other senses. For this purpose, it is well to adopt the distinction made by SPURZHEIM and others, of the functions of the senses into *immediate* and *mediate*. Each sense has its immediate function, which it possesses exclusively; for which, in other words, no other can be substituted. The touch instructs us regarding temperature; the taste appreciates savours; the smell, odours; audition, sound; and vision, colors. These are the *immediate* functions of the senses, each of which can be accomplished by its own organs, but by no other. As concerns the immediate functions of the senses, therefore, the touch can afford no correction. Its predominance as regards the *mediate* functions of the senses is likewise exaggerated. The *mediate* functions are those that are auxiliary to the senses, consisting in the impressions they furnish to the mind and by aid of which it acquires its notions of bodies. The essential difference between those two sets of functions is, that the mediate can be effected by several senses at once. Vision, olfaction, and audition participate in judging of distances, as well as the touch; the sight instructs us regarding shape, &c. It has, indeed, been affirmed by metaphysicians, that the touch is necessary to several of the senses to give them their full power; that we could form no notion of the size, shape, and distance of bodies, unless instructed by this sense. The remarks already made, have proved the inaccuracy of this opinion. The farther examination of it will be resumed under the subject of vision. The senses are, in truth, of mutual assistance. If the touch falls into error, as in the case of inaccurate appreciation of temperature, the sight, aided by appropriate instruments, dispels it. If the fingers, crossed, convey to the brain the sensation of two rounded bodies, when one only exists, the sight apprizes us of the error; and if the sight and touch united impress us with a belief in the identity of two liquids, the smell or the taste will often detect the erroneous inference.

On the whole then we must conclude that the senses mutually aid each other in the execution of certain of their functions; but that each has its province, which cannot be invaded by any of the others; and that too much preponderance has been ascribed to the touch by metaphysicians and physiologists. Administering, however, so largely to the mind, it has been properly ranked with vision and audition as an intellectual sense."

We make the following extract from the remarks of the author upon the sense of taste, on account of the ingenious hypothesis with which it concludes, and we do it the more willingly, as it conveys an important principle in hygiene, which cannot be too often impressed upon the mind of the young practitioner.

"To appreciate a savour accurately, the sapid substance must remain for some time in the mouth: when rapidly swallowed the impression is extremely feeble, and almost null. Of this fact we take advantage, when compelled to swallow any nauseous substance: whilst we retain a savory article long in the mouth, in order that we may draw from it all its sweets. How different, too, is the consent of the auxiliary organs under these two circumstances. Whilst a luscious body augments the secretion of the salivary glands, or cause the mouth "to water," as it has been called, projecting the saliva, at times, to a distance of some feet from the mouth, and disposing every part to approach and mingle with it; a nauseous substance will produce constriction of every secretory organ, and this effect will extend even to the stomach itself, so that it will often reject the offending article as soon as it reaches the cavity. We can thus understand how, *cæteris paribus*, an article, which is pleasing to the palate, may be more digestible than one which excites disgust, and *vice versa.*"

There is generally supposed to be something mysterious and inexplicable upon the subject of antipathies, as to the manner that the subjects of them are made sensible of the vicinity of the offending animal, apparently without the assistance of any of the senses; certainly without any conscious impression being made upon them. Dr. D. refers it to a preternatural delicacy of smell, and the cases which he gives in illustration add great force to the opinion.

"To the same cause must be ascribed the delicacy of olfaction, generally observed in the blind. The boy MITCHELL, who was born blind and deaf, and whose case will have to be detailed at length hereafter, was able to distinguish the entrance of a stranger into the room by the smell alone. A gentleman, blind from birth, from some unaccountable impression of dread or antipathy, could never endure the presence of a cat in the apartment. One day, in company, he suddenly leaped up, got upon an elevated seat, and exclaimed that there was a cat in the room, begging them to remove it. It was in vain that the company, after a careful inspection, assured him he was under an illusion. He persisted in his assertion and state of agitation when, on opening the door of a small closet in the room, it was found that a cat had been accidentally shut up in it."

One of the most learned chapters in the work before us is upon the *sense of hearing*. Yet such is the obscurity attending the physiology of this important sense, that the chief interest of this part consists in the anatomical details, and the recapitulation of the ingenious hypotheses, which have been formed to explain the manner in which the human ear appreciates sounds.

One of the most interesting inquiries connected with the physiology of hearing is, whether the faculty of appreciating musical sounds depends upon the conformation of the ear, or it is strictly an intellectual faculty. Dr. D. adopts the latter opinion, and supports it by the following train of reasoning.

"The man who is totally devoid of musical ear, hears the sound distinctly. His sense of hearing may be as acute as that of the best musician. It is his appreciation that is defective. He hears the sound, but is incapable of communicating it to others. The organ of appreciation is in this, as in every other sense,—the brain. The physical part of the organ may modify the impression, which has to be made upon the nerve of sense:—the latter is compelled to transmit the impression as it receives it; and it is not until the brain has acted, that perception takes place, or that any idea of the physical cause of the impression is excited in the mind. If, from faulty organization, such idea is not formed in the case of musical tones, the individual is said not to possess a musical ear. But the fault lies in his cerebral conformation. We do not observe the slightest relation between musical talent and delicacy of hearing. The best musicians have not necessarily the most delicate sense, and, for the reason already assigned, it will be manifest, why the idiot, whose hearing may be acute, is incapable of singing, as well as of speaking. Again, we do not see the least ratio in animals between the power and character of their music, and the condition of their auditory sense. We are compelled then to admit, that the faculties of music and speech are dependent upon the organization of the brain; that they require the ear as a secondary instrument; but that their degree of perfection is by no means in proportion to the delicacy of the sense of hearing."

The faculty is certainly improved very much by education.—The aptitude, however, is laid in cerebral organization, and is developed by the education of the instrument, the ear, as well as the encephalic, or intellectual organ, without which no such appreciation could be accomplished.

The physiology of none of the senses, indeed, of none of the functions, is possessed of such general interest as that of vision. The importance of the sense itself, the interest of the science of optics with which it is so intimately connected, the beautiful and perfect adaptation of the instrument by which vision is effected, together with the obvious use of all the parts in its complicated structure, render it a study of peculiar interest, not only to the physician, but to the general reader. Into this subject the author enters at great length, presenting to his reader every thing of importance that can be collected either from reading or observation whether of a scientific or practical nature.

One of the most interesting points in the mechanism of the eye, is the fact that it constitutes an achromatic lens. This important fact, which is too generally omitted in the popular works on physiology, is very fully and clearly illustrated by the author.

The rays of light proceeding from a point and falling upon a convex lens are caused again to converge to a point, and thus a complete image will be formed of any object. It is this image of which the eye takes cognisance in vision. The rays of light, however, by the action of the lens are divided into their primitive colors, and as some are more refrangible than others, those which are the most so are brought to a focus, sooner than the rest; hence arises an indistinctness of the image, a confusion of the lights and colors, which confounds its proportions. In the construction of optical instruments, the imperfection arising from this source has been avoided by the following expedient. It has been ascertained that the dispersive and refractive powers of the same medium are not always strictly proportionate to each other; some substances which refract the light very powerfully, have little tendency to separate it into its elementary rays, and, vice versa, others disperse the rays very much, while they refract them but little. By employing, therefore, a convex lens with a great refractive and a very slight dispersive power, and counteracting it by a concave lens with a slight refractive but a very great dispersive power, the difficulty arising from the dispersion of the rays will be obviated, without preventing them from forming an image.

This arrangement is supposed to have been made in the human eye, in which there are three lenses, presenting three convex and two concave surfaces. The aqueous humor whose refractive power is not very considerable, is a concavo-convex lens—the chrystalline, a double convex—and the vitreous presents a single concave surface. The chrystalline is more refractive, and the other two humors are supposed to have a greater dispersive power. Thus the human eye is an achromatic optical instrument.

An important question here presents itself for consideration—what is the effect of couching the chrystalline lens upon the focal distance of the eye? It generally, we believe, increases the focal distance very considerably, so that after this operation, a very convex lens is necessary to distinct vision. This fact proves that the lens has a much greater refractive power than either of the other humors, for as the surfaces lie, of necessity, in close contact, the concave surfaces of the humors must accurately correspond with the convex surfaces of the lens. If, therefore, their powers were equal, the removal of the lens would have no effect upon the focal distance of the eye.

The manner in which the eye accommodates itself to the distance of objects, has been a source of great perplexity to opticians. The following appears to us to be the true state of the case.

"Sir CHARLES BELL conceives "that the mechanism of the eye has not so great a power of adapting the eye to various distances as is generally imagined, and that much of the effect attributed to mechanical powers, is the consequence of the motion of the pupil, the effect of light and of attention. An object looked upon, if not attended to, conveys no sensation to the mind. If one eye is weaker than the other, the object of the stronger eye alone is attended to, and the other is entirely neglected: if we look through a glass with one eye, the vision with the other is not attended to." "The mind," he adds, "not the eye, harmonizes with the state of sensation, brightening the objects to which we attend. In looking on a picture or panorama, we look to the figures, and neglect the back ground; or we look to the general landscape, and do not perceive the near objects. It cannot be an adaptation of the eye, but an accommodation and association of the mind with the state of the impression.

The view, we have expressed upon the subject, is strikingly confirmed by the calculations of M. DE SIMONOFF, a learned Russian astronomer, who asserts, that from a distance of four inches to infinity, the changes in the angle of refraction do not exceed twenty-three minutes, so that the apices of luminous cones, in a properly formed eye, must always fall within the substance of the retina, and hence no variation in the shape of the eye, according to the distance of the object, can be necessary."

The views of M. De Simonoff can be easily confirmed by a very simple experiment. Let a lens with a focal distance of about one inch, he placed before a small aperture in a darkened room. If a screen be now placed at the focal distances, it will be found that images of objects, correct and well defined in their outlines, will be formed, though their distance should vary from four inches to an indefinite space. Within this range, the eye, assisted as it is by memory and the imagination, is able to recognise very distinctly, objects that are familiar to it, although the range of clear and distinct vision for objects which are not familiar to us, is more contracted than most persons are aware of. Few persons can see as well at four inches as they can at twelve or eighteen; and when objects are brought nearer for accurate investigation, it is rather for the purpose of increasing the intensity of the light, than the distinctness of the image.

In treating of the eye, has not the author, in speaking of the "aberration of sphericity," fallen into an error regarding the true shape of the eye? The curve of the eye is not spherical but parabolic, and, consequently, is not, we presume, subject to the aberration of sphericity.

It is frequently asked, why, as an impression is made upon each of the eyes, we do not see objects double? For the same reason, we imagine, that we do not feel objects double when we grasp them with both hands, and for the same reason, probably, that we have a distinct idea of the unity of an object which addresses itself to all the senses at once.

There has been a similar difficulty experienced in accounting for the fact, that, although the image of an object of sight, is inverted upon the retina, we see it in its true position. The explanation probably is to be found in the fact, that in looking at an object, we do not embrace the whole at one view, but examine the different parts in succession. For this purpose, we insensibly move the eye so as to bring the different parts of the object upon the most sensible part of the retina, and we probably form our idea of its position, not from the image upon the retina, but from the motion of the eye.

The following remarks, pointing out an anomaly in Mr. Charles Bell's System of the Nerves, are worthy of being repeated.

"The different distributions of the motor nerves of the eye have been described in the anatomical sketch. It is there stated, that the superior oblique muscle receives one whole pair of nerves:—the fourth pair. This nerve, then, it seemed to Sir Charles Bell, must be concerned in the functions we have described; and as the various involuntary motions of the eyeball are intimately concerned in expression, as in bodily pain, and in mental agony,—in which the action of the direct muscles seems, for a time, to be suspended,—he was led to consider the fourth pair as a nerve of expression—a respiratory nerve; and, hence, intimately connected with the facial nerve of the seventh pair, which, as has already been remarked, is the great agent in the twinkling of the eyelids. Anatomical examination confirmed this view;—the roots of the nerve being found to arise from the same column as the other respiratory nerves. The coincidence of this twinkling and of the motion of the eyeball upwards was, therefore, easily understood.

There is a difficulty, however, here which has doubtless already suggested itself. The fourth pair of nerves is distributed to the superior oblique only; the lesser oblique receives none of its ramifications. They cannot, therefore, be identically situated in this respect. Yet they are both considered by Sir Charles Bell as involuntary muscles. The action, indeed, of the lesser oblique would appear to be even more important than that of the greater oblique, as the function of the latter, when acting singly, is to carry the eye upwards and inwards; and, when the action of its antagonist is abolished, this is more clearly manifested. Sir Charles Bell found, that the effect of dividing the superior oblique was to cause the eye to roll more forcibly upwards: in other words, it was given up, uncontrolled, to the action of the antagonist muscle.

This difficulty, although it is not openly stated by Sir Charles, must have impressed him; as, after having referred to the effect of the division of the superior oblique, he is constrained to suggest an influence to the fourth pair, which would, we think, be anomalous:—that it may, on certain occasions, cause a relaxation of the muscle to which it goes, and, in such case, the eyeball must be rolled upwards!

We have still, therefore, much to learn regarding this subject, into which so much interest, and, at the same time, so much uncertainty has been infused."

The whole chapter on vision is a very interesting one, evincing on the part of the author a great deal of research and learning, and a familiar acquaintance with all that has been written upon the physiology of vision, or the science of optics.

A very considerable chapter is devoted to the consideration of the mental faculties, to which the author has given a prominence of position, that few writers on physiology have considered them entitled to in a work of this kind. In this part of the work he has given a pretty full exposition of the opinions of Gall and Spurzheim, with his objections at length to their system. Being but slightly acquainted with the opinions of these gentlemen, we are not able to pronounce upon the merit of the remarks by which they are attempted to be controverted. But, although not very much predisposed to favor their views, we do not think that the author's objections to them are satisfactory, or that they will be considered by the friends of phrenology, as giving a statement so fair as was the duty of the author of a work like the present.

The chapter on muscular motion embraces a great variety of subjects, and exhibits in a very favorable point of view the extent and accuracy of the author's researches into every department of study, necessary to illustrate the science of which he is treating.

Great pains have been devoted of late years to ascertain the precise part of the brain which forms the seat of volition. Although these researches have been for the most part fruitless, or at least have led to very indefinite results, yet they have, nevertheless, like the search for the "Philosopher's Stone," served to develope many curious and interesting facts, that will probably become hereafter the groundwork of important physiological principles. Among these, one of the most interesting is the discovery which Magendie supposes himself to have made, and of which an account is given in the following extract:

The experiments of Magendie, on this subject, are pregnant with important novelty. We have already referred to those which concern the cerebral hemispheres and cerebellum, as the encephalic organs of the general movements, in the mode suggested by Rolando and Flourens, and others. In addition, he affirms, 'that there exist, in the brain, four spontaneous impulses or forces, which are situated at the extremity of two lines, cutting each other at right angles; the one impelling forwards; the second backwards; the third from right to left, causing the body to rotate; and the fourth from left to right, occasioning a similar movement of rotation.' The first of these impulses he fixes in the cerebellum and medulla oblongata; the second in the corpora striata; and the third and fourth in each of the peduncles of the cerebellum.

"1. *Forward Impulse.*—It has often been observed by those who have made experiments on the cerebellum, that injuries of that organ cause the animals to recoil, and manifestly against their will. Magendie asserts, that he has frequently seen animals wounded in the cerebellum, make an attempt to advance, but be immediately compelled to run back; and he says, that he kept a duck for eight days, the greater part of whose cerebellum he had removed, which did not move forwards during the whole of that time, except when placed upon water. Pigeons into whose cerebella he thrust pins, constantly walked, and flew backwards, for more than a month afterwards. Hence, he concludes, that there exists either in the cerebellum or medulla oblongata, a force of impulsion, which tends to cause animals to go forwards.

"2. *Backward Impulse.*—When the corpora striata are removed, MAGENDIE found that the animal darted forward with great rapidity; and, if stopped, still maintained the attitude of running. This was particularly remarked in young rabbits; the animal appearing to be impelled forward by an inward and irresistible power; and passing over obstacles without noticing them. These effects were not found to take place, unless the white radiated part of the corpora striata was cut: if the grey matter was alone divided, no modification was produced in the movements. If only one of the corpora was removed, it remained master of its movements, and directed them in different ways; stopping when it chose; but, immediately after the abstraction of the other, all regulating power over the motions appeared to cease, and it was irresistibly impelled forwards.

"3. *Lateral Impulse.*—Again, if the peduncles of the cerebellum —the crura cerebelli—be divided in a living animal, it immediately begins to turn round, as if impelled by a considerable force. The rotation or circumgyration is made in the direction of the divided peduncle; and, at times, with such rapidity that the animal makes as many as sixty revolutions in a minute. The same kind of effect is produced by any vertical section of the cerebellum, which implicates, from before to behind, the whole substance of the medullary arch, formed by that organ above the fourth ventricle, but the movement is more rapid, the nearer the section is to the origin of the peduncles; in other words, to their point of junction with the pons varolii.

MAGENDIE affirms, that he has seen this movement continue for eight days, without stopping, and, apparently, without suffering. When any impediment was placed in the way, the motion was arrested; and, under such circumstances, the animal frequently remained with its paws in the air and ate in this attitude. What he conceives to have been one of his most singular experiments was, the effect of the division of the cerebellum into two lateral and equal halves; when the animal appeared to be alternately impelled to the right and left, without retaining any fixed position. If he made a turn or two on one side, he soon changed his motion and made as many on the other.

M. SERRES, who is well known as a writer on the comparative anatomy of the brain, and must have unusual opportunities for observation at the Hospital *La Pitié*, to which he is attached—gives the case of an apoplectic, who presented, amongst other symptoms the singular phenomenon of turning round, like the animals in the experiments just described; and on dissection, aa apoplectic effusion was found in this part of the encephalon.

"On dividing the pons varolii vertically, from before to behind,

MAGENDIE found that the same rotary movement was produced; when the section was to the left of the median line, the rotation was to the left, and *vice versa;* but he could never succeed in making the section accurately on the median line."

Other experimenters again have attempted to prove that the anterior portion of the corpora striata presides over the movements of the lower limbs, and the optic thalamus over those of the upper. Upon this subject the author makes the following remarks.

"It is sufficiently obvious, from the whole of the preceding detail, that the mind must still remain in doubt, regarding the precise part of the encephalon engaged in the functions of muscular motion. The experiments of MAGENDIE are, perhaps, more than any of the others, entitled to consideration. They appear to have been instituted without any particular bias; to subserve no particular theory; and they are supported by pathological facts furnished by others. M. MAGENDIE is, withal, an accurate and practised experimenter, and one to whom physiology has been largely indebted. His vivisections have been more numerous, perhaps, than those of any other individual. His investigations, however, on this subject clearly show, that owing to the different structures of animals, we cannot draw as extensive analogical deductions from comparative anatomy and physiology as might be anticipated. The greatest source of discrepancy, indeed, between his experiments and those of MM. ROLANDO and FLOURENS, appears to have arisen in the employment of different animals. Where the same animals were the subject of the vivisections, the results were in accordance. The experiments demand careful repetition, accompanied by watchful and assiduous observation of pathological phenomena; and, until this is effected, we can, perhaps, scarcely feel justified in deducing from all these experiments and investigations, more than the general propositions, regarding the influence of the cerebro-spinal axis on muscular motion, which we have already enunciated."

One of the most interesting parts of physiology is, the application of mechanical laws to muscular motion. The power and tenacity of the muscles, when we take into consideration their structure, and weakness after death, is astonishing. Every where, in the formation of the limbs, we observe that power is sacrificed to elegance and convenience of form, and to rapidity of motion. The muscles most favorably situated for mechanical power cannot exert more than one fiftieth part of their power in the erection of a weight. The following are some of the modes by which the power of the muscles is diminished.

1. The insertion of the muscle into the bone at a point much nearer the fulcrum, or the centre of motion, than that upon which the power acts. From this circumstance the deltoid muscle, which is more favorably situated than most of the other muscles, makes but one third of its strength effective in elevating the weight.

2. The obliquity of the angle of insertion. The loss of power by this in many of the muscles is very great.

3. The oblique position of the fibres of the pennated muscles.

4. Muscles which coincide in action are frequently inserted into the bone in such a manner, as, in a very considerable degree, to counteract each other's influence.—As for instance the latissimus dorsi and pectoralis major in bringing the arm to the side.

5. It was supposed by the earlier writers, and has since been generally copied by succeeding authors that there is a loss of half the power, when the muscle is inserted between the fulcrum and the power. This, however is evidently an error, the loss being always proportionate to the comparative distance from the fulcrum, of the power and the weight.

Lastly much force is spent when the muscles pass over many joints—the antagonist muscles exert, in many cases, a considerable counteracting force, and probably when very great contraction takes place the action of the muscle is impeded by the fascia which invests it. It has been generally supposed by anatomists that the tendency of the fascia is to strengthen the muscle. It is difficult to conceive, however, how an effect of this kind can be produced. A more rational use would appear to us to be, the protection of the muscle, its tendon, or the joint, against the effect of sudden or violent exertion of muscular strength, which sometimes takes place; as, for instance, when the muscles are spasmodically contracted to prevent the body from falling. In support of this opinion we may remark that in those muscles which are not provided with this fascia, rupture of the tendon frequently takes place, and they are, in addition, peculiarly liable to cramps after over-action, as is the case with the flexors of the leg; while on the other hand in the muscles of the thigh which are subjected to equally violent and long continued action, these results are scarcely ever produced.

These disadvantages, however, are more than compensated by the benefits which result from this arrangement.

"On the other hand, there are certain arrangements, which augment the power developed by muscles; as the thick articular extremities of the bones; the patella and sesamoid bones in general; all of which enlarge the angle, at which the tendon is inserted into the bone or lever. The projecting processes for muscular attachments, as the trochanters, the protuberance of the os calcis, the spinous processes of the vertebræ, &c. augment the arm of the lever and are, thus, inservient to a like valuable purpose. The smoothness of the articular surfaces of bones, tipped as they are, with cartilage; and the synovia, which lubricates the joints, by diminishing the friction, also augment the power, as well as the bursæ mucosæ, which are interposed, whereever there is much pressure or friction. The trochleæ or pulleys act only in directing the force, without augmenting its amount; and the same may be said of the bony canals and tendinous sheaths, by which the tendons of the muscles, especially those passing to the fingers and toes are kept in their proper course.

"Still, it must be admitted, that, as regards the effort to be exerted by the muscles, it must, in almost all cases, be much greater than the resistance it has to overcome. The very fact of the lever of the third kind being that which prevails in our movements exhibits this. The mere mechanician has conceived this to be an unwise construction; and that there is a needless expense of force for the attainment of a determinate end. In all cases we find, that the expense of power has been but little regarded in the construction of the frame; nor is it necessary that it should have been. It must be recollected, that the contraction of the muscle is under the influence of volition, and that within certain limits, the force to be employed, is regulated by the influx sent by it into the muscles. The great object in the formation of the body, appears to have been;—to unite symmetry and convenience, with the attainment of great velocity and extent of motion, so that, whilst the power is moving through but a small space, the weight or resistance shall move rapidly through one more extensive. We have seen that, in these respects, the lever of the third kind is most fitting. With any other, indeed, less power might be required; but we should have less extent of motion and less velocity, whilst the symmetry and convenience of the body would be destroyed."

Besides the advantages enumerated by the author, there are other compensations which obviate very considerably the loss of power produced by the unfavorable insertion and position of the muscles.

The muscle in elevating a weight placed at the extremity of the limb, does not raise the weight directly upward, but causes it to describe an arc of a circle, by which upon a principle in mechanics—that of the inclined plane—it gains in power in the ratio that exists between the circumference of the circle and the diameter—rather more than three to one. Upon the same principle the degree of force necessary to keep the body in the erect position is much less than would be necessary, if the body were to be borne directly upwards. The body does not require one tenth of the muscular power to preserve it in the erect position, that is necessary to raise it to that position, because its actual descent while in that position, is very small in proportion to the arc of the circle, which the bone describes. It is upon this principle that the gastrocnemii muscles, notwithstanding their oblique insertion into the tibia, are enabled to sustain the weights which are sometimes placed upon the body.

A great deal of labor has been spent in attempting to calculate the precise degree of power possessed by the muscles. The fruitlessness of such attempts may well be inferred from the discrepancy which prevails among the experimenters who have attempted the calculation. Upon this subject Dr. D. makes the following judicious observations.

"In executing the complex movements of any part of the frame, a combination of the action of the different muscles, attached to the part, generally occurs, rendering the process one of a complicated character. This, if no other cause existed, would render it extremely difficult to calculate the precise degree of force, which particular muscles, alone or in combination, are capable of exerting. The mathematical physiologists made multifarious attempts in this direction; but their conclusions were most discrepant. When we bear in mind, that the force, capable of being exerted by any muscle, is dependant upon the proper organization of the muscle, and likewise upon the degree of energy of the brain, it will be apparent, that all attempts of this kind must be futile. We can determine, with nicety, the effect of which the parts are capable, supposing them inanimate structures. We can calculate the disadvantages, caused by the insertion of the power near the fulcrum; by the obliquity of the line of action of the power, &c.; but we have not the slightest data for estimating the effect, produced by the nervous influx,—by that mysterious process, which generates a new force, and infuses it into the muscles, in a manner so unlike that in which the ordinary mechanical powers are exerted. The data, necessary for such a calculation, would be the precise influx from the brain,—the irritability of the muscle,—the mechanical influences, dependant on the straight or oblique direction of the fibres composing the muscle, as regards the tendon,—the perpendicular or oblique direction in which the tendon is attached to the bone,—the particular variety of lever,—the length of the arm of the power and that of the resistance,—the loss sustained from friction, and the diminution of such loss caused by the cartilages, that tip the bones, and by the synovia, &c.—data, which it is impossible to acquire; and hence the solution of the problem is impracticable.

One great source of the combination of muscular motion is, the necessity for rendering one of the attachments fixed, in order that the full force may be developed on the other. In but few of the muscles is the part whence the muscle originates, steady. To these few the muscles of the eye belong, which arise from the inner part of the orbit and pass forward to be inserted into the organ. To show how very distant muscles may be concerned in this fixation of one end of a muscle, when it is excited to the development of plenary power, we will take the case of the deltoid. This muscle arises from the scapula and clavicle, and is inserted into the os humeri: but the scapula and clavicle, themselves, are not entirely fixed; and accordingly, if the deltoid were to contract alone, it would draw down the scapula and clavicle, as well as elevate the humerus. If, therefore, it be important to produce the latter effect only, the scapula and clavicle must be fixed by appropriate muscles; as by the rhomboidei, trapezius, &c. These muscles, however, arise from various vertebræ of the neck, which are themselves movable. It becomes necessary, therefore, that the neck should be fixed by its extensors, which arise from the lumbar and dorsal regions. By the united action of all these muscles, the deltoid is able to exert its full effect, in elevating the humerus. But the deltoid, like other muscles, is capable of acting inversely; as in the case of a person lying on the ground, and attempting to raise himself, by laying hold of any object above him. The hand and forearm are thus rendered firm, and the deltoid now contracts from origin to insertion, and, consequently, elevates the scapula and clavicle.

Again, if a person, in the recumbent posture, endeavor to bend the head forwards, the recti muscles of the abdomen are firmly contracted, for the purpose of fixing the sternum, whence the sterno-cleido-mastoidei muscles in part arise, which can then exert their full power in bending the neck forwards.

These instances will be sufficient to exemplify the mode, in which the muscular motions are combined. The same principle prevails over the whole body; and where a great number of parts has to be moved, the case must, necessarily, be still more complex."

In treating of the voice Dr. D. has entered not only into the physiology of his subject, and examined very fully and ingeniously the different opinions of physiologists upon this obscure and intricate function, but he has also entered at some length upon the philosophy of language, and, perhaps, invaded provinces to which he has no legitimate claim.

In accounting for the voice, the author is disposed to look upon the opinion of Savart, as having the strongest claims to our notice. For a full account of this theory we refer our readers to the author. For the benefit of those who may not have this in their power, the following quotation will give a general idea of his views.

"These different physical conditions SAVART invokes to account for the different tones of the human voice,—under the theory, that the vocal organ, composed of the larynx, pharynx, and mouth, forms a conical tube, in which the air is set in vibration by a movement similar to that which prevails in organ pipes.

The trachea is terminated by a cleft,—the glottis,—which is the inferior aperture of the vocal instrument. This cleft, which is capable of being rendered more or less narrow, plays the same part as the *lumiere des tuyaux a bouche*, or the narrow space in the organ pipe, at the edge of the *biseau* or *languette**, along which the air passes. The air clears it, traverses the ventricles of the larynx, or the cavity of the instrument, and strikes the superior ligaments. These surround the upper aperture of the instrument, and fulfil the same function as the *biseau* of the organ pipe. The air, contained in the larynx, now vibrates, and sound is produced. This sound acquires intensity, because the waves that constitute it are extended into the vocal tube, situated above the larynx, and excite in the column of air filling it, a movement similar to that occasioned in the tube of a flute; except that the tone can be much varied, because the larynx being a short tube, can give rise to various tones by simple modification in the velocity of the air sent through it; and, moreover, the vocal tube has the same power, its parietes being membranous, of a vibratory nature, and capable of different degrees of tension. The inferior or outer part of the vocal tube is equally constituted of elastic parietes,

* The *biseau* or *languette* is the diaphragm, placed between the body of an organ pipe and its foot.

susceptible of varied tension; and the mouth, by modifying the dimensions of the column of air within the vocal tube, exerts an influence on the number of vibrations, which the column is capable of experiencing; whilst the lips can convert the channel at pleasure into an open or closed conical tube. Certain sounds, SAVART affirms, are produced altogether in the ventricles of the larynx,—those of pain and the *falsetto* voice, for example. They can be elicited, even when the vocal tube has been removed; and there are animals, in which the vocal organ is reduced to the ventricles of the larynx—frogs for instance. SAVART, consequently, considers, that the human vocal organ has, in its essential parts, a striking analogy to the action of the bird-call; and, in this way, he explains the use of the superior ligaments, which are entirely overlooked in the different theories of the voice previously propounded.

We have given Savart's view at some length, in consequence of its ingenuity, and of its seeming to explain better than any other theory the varied tones of which the human voice is susceptible. It cannot, however, be esteemed established, inasmuch as it is diametrically opposed in many points, to the observations and vivisections of other distinguished physiologists; who, it has been seen, affirm, that voice is produced solely by the inferior ligaments; that all parts above these may be destroyed, and yet voice may continue; and that, a wound in the ventricles, which permits the exit of air through the parietes of the larynx, does not destroy the function. Our notions on this point must not, therefore, be considered definite. Farther experiments are necessary; and, in all the deductions from them, great importance will have to be attached to the vital action of the parts; especially of the intrinsic muscles, which are capable of modifying the situation of parts, and the character of the function in myriads of inappreciable ways.

The following calculations respecting the tone of the voice are sufficiently curious to warrant their insertion.

"*Tone of the voice.*—Nothing can exceed the human organ of voice in variety and execution. Dr. BARCLAY has endeavored to calculate the different changes of which it is susceptible, proceeding on the principle that where a number of movable parts constitute an organ, destined to some particular function; and where this function is varied and modified by every change in the relative situation of the movable parts, the number of changes, producible in the organ, must at least equal the number of muscles employed, together with all the combinations of which they are capable. The muscles, proper to the five cartilages of the larynx, are, at least, seven pairs; and fourteen muscles, that can act separately or in pairs, in combination with the whole or with any two or more of the rest, are capable of producing upwards of sixteen thousand different movements,—not reckoning as changes the various degrees of force and velocity, with which they are occasionally brought into action. These muscles, too, are only the proper muscles of the larynx, or the muscles, restricted in their attachments to its five cartilages. They are but a few of the muscles of voice. In speaking, we use a great many more. Fifteen pairs of different muscles, attached to the cartilages, or to the os hyoides, and acting as agents, antagonists, or directors, are constantly employed in keeping the cartilages steady, in regulating their situation, and moving them as occasion requires,—upwards and downwards, backwards and forwards, and in every intermediate direction, according to the course of the muscular fibres or in the diagonal between different fibres. These muscles independently of the former, are susceptible of upwards of 1073,841,800 different combinations; and, when they co-operate with the seven pairs of the larynx, of 17592186,044,415; exclusive of the changes, which must arise from the different degress of force, velocity, &c. with which they may be brought into action.

But these muscles are not the whole, that co-operate with the larynx, in the production of the voice. The diaphragm, the abdominal muscles, the intercostals, and all that directly or indirectly act on the air or on the parts to which the muscles of the glottis or os hyoides are attached,—in short, all the muscles, that receive nerves from the respiratory system of Sir CHARLES BELL,—contribute their share. The numerical estimate would consequently require to be largely augmented. Such calculations are, of course, only approximate, but they show the inconceivable variety of movement of which the vocal apparatus is directly or indirectly susceptible.

As the author's remarks upon *artificial language* appear to us to contain some important errors, which pervade almost every work upon the subject in the language, we shall venture with great deference to universally received opinions, to attempt to controvert them. For this purpose, we make a number of detached quotations upon which we wish to offer a few comments.

"In the preceding enumeration of the simple sounds, constituting the alphabet, C, Q, W, X and Y, have been excluded, for the following reasons. C has always the sound of either S or K, as in *cistern* or *consonant*. Q has the sound of *koo*, as in quart, (*kooart;*) W of *oo*, as in word, (*oourd;*) X of *ks*, or Z, as in vex, (*vecks,*) or Xerxes, (*zerkses;*) whilst Y has the sound of I or E, as in wry or yard, (*wri* or *eeard.*) *Ch*, *Sh*, and *Th*, have been added, as being true alphabetic or simple sounds.

"Letters have been usually divided into two classes,—*vowels* and *consonants*.

"The *vowels* or *vocal sounds* are so called, because they appear to be simple modifications of the voice, formed in the larynx, uninterrupted by the tongue and lips, and passing entirely through the mouth.

Such at least is the case with those that are reckoned *pure vowels.* These, in the English alphabet, are five in number,—A. E, I, O, and U. W and Y are, likewise, vowel sounds in all situations.

"In enunciating A, as in *fate*, the tongue is drawn backwards and slightly upwards, so as to contract the passage immediately above the larynx. In sounding E, the tongue and lips are in their most natural position, without exertion. I is formed by bringing the tongue nearly into contact with the bony palate. O, by the contraction of the mouth being greatest immediately under the uvula, the lips being also somewhat contracted; and, in the production of U, the contraction is prolonged beneath the whole of the soft palate.

"The consonants are usually divided into *mutes*, *semi-vowels*, and *liquids*.

"The *mutes* are such as emit no sound without a vowel; as *b, p, t, d, k*, and *c* and *g* hard.

"The *semi-vowels* are such as emit a sound, without the concurrence of a vowel, as *f, v, s, z, x, g* soft or *j*.

"The *liquids* are such as flow into, or unite easily with, the mutes, as *l, m, n, r*. These letters issue without much obstacle; and hence perhaps their name.

"In tracing the mode in which the different consonants are articulated, we find, that certain of them are produced by an analogous action of the vocal tube; so that the physiology of one will suffice for the other also. For instance, the following nearly correspond;—

| | | | | | |
|---|---|---|---|---|---|
| *p* | *f* | *t* | *s* | *k* | *ch* |
| & | & | & | & | & | & |
| *b* | *v* | *d* | *z* | *g* | *j*. |

B and P are produced when the lips, previously closed, are suddenly opened. B differs from P in the absence, in the latter, of an accompanying vocal sound. F and V are formed by pressing the upper incisor teeth upon the lower lip. They are, consequently, not well enunciated by the aged, who have lost their teeth. F differs from V only in the absence of an accompanying vocal sound. T and D are formed by pressing the tip of the tongue against the gums behind the upper incisor teeth: D is accompanied by a vocal sound; T not. S and Z are produced by bringing the point of the tongue nearly in contact with the upper teeth, and forcing the air against the edges of the teeth with violence. S differs from Z in the absence of the vocal sound. K and G are formed by pressing the middle of the tongue against the roof of the mouth, near the throat; and separating the parts a little more rapidly to form the first, and more gently to form the last of those letters. In K, the accompanying vocal sound is absent. Ch and J are formed by pressing *t* to *sh;* and *d* to *zh.* In Ch, there is no accompanying vocal sound."

Having been induced, from personal considerations, to pay a good deal of attention to the physiology of the voice, and particularly to the enunciation of the letters, we have been led to form some opinions very much at variance with those generally taught in the schools.

The distinction generally made between the vowels and the consonants, appears to us peculiarly defective. To form a correct opinion upon this subject, we are to recollect that the organs of speech are composed of, *the proper organ of sound*, and *the organs of articulation.* The former by the universal consent of physiologists, is placed the larynx—the latter are, the lips, teeth, tongue, palate, and the palatine arches. The vowels are laryngeal sounds, modified by the action of the breath upon the organs of articulation—the consonants are formed by the action of the air upon the organs of articulation alone. With what propriety Dr. D. can say that "the vowels are so called, because they appear to be simple modifications of the voice formed in the larynx, and uninterrupted by the tongue and lips," we cannot conceive; since he immediately describes the action of the organs of articulation in modifying the purest vowels, a, e, i, o, and u. The organs of articulation are necessary to the pronunciation of both. The difference between them is this; in pronouncing the vowel the air acts simultaneously upon the organ of sound and the organs of articulation—while in pronouncing the consonant it acts upon the organs of articulation alone and the vocal sound is not produced until the consonant is completed.

There is this further difference that the vowels may be prolonged for an indefinite length of time, the consonants are incapable of prolongation. The general opinion is that the consonants cannot be sounded without the aid of a vowel—that the semivowels have an imperfect sound, which admits of prolongation—while the mutes cannot be sounded at all. After close and repeated observations upon this subject, we are inclined to the opinion that both mutes and semivowels admit of a distinct and audible sound, without the concurrence of the vocal organ, and that examples of this may be witnessed every day in the first attempts of the infant to speak. If this opinion be true, the semivowels are no more capable than the mutes of a prolonged sound, both being produced by the sudden explosion of breath upon the organ of articulation; and the only difference between them will be, that in the case of the former the organ of articulation retains the situation in which it was placed, when the sound was produced, and by its action upon the breath still keeps up a slight sound—which, however, is very different from the true sound of the consonant, while in sounding the consonant the position of the organ is immediately changed and it is therefore incapable of any prolongation whatever. We are the more inclined to adopt this opinion, from observing that the organs of articulation are certainly capable of producing very distinct and audible sounds without the concurrence of the larynx; as is the case in whistling, &c. and we think ourselves justified in coming to the conclusion that the difficulty of producing isolated consonant sounds arises from the universal practice, in speech, of accompanying them with the vowels which are indeed necessary to give them sufficient body to be heard at any distance.

Whether this view of the case be correct or not, it is still true, that the mutes, without the concurrence of a vowel, emit as distinct a sound, as the semivowels, and that the only difference between them exists in the former being incapable of prolongation.

We before remarked that the sound of the vowels is modified by the organs of articulation; and it is, probably, by the different position of these organs, that all the varieties of the vowel sounds are produced. We may also remark, that the position of the organs of articulation, in sounding some of the consonants is the same that it is in pronouncing certain of the vowels. This is the case with the consonant w and vowel sound oo. This coincidence has led Dr. D. to adopt the opinion that the two sounds are identical, and accordingly he has expunged the w from the list of elementary sounds. In this we think he is most clearly in an error. The difference between them consists in the fact, that in w, the effort is made upon the organ of articulation alone, while in the oo, the vocal organs act in concert with the organs of articulation. A little attention to this circumstance, we think, will bring every one to the conclusion that w is a consonant.

The same remark will apply to y, which Dr. D. is disposed to consider as a vowel, under all circumstances. Y has two sounds, the sound of i—a vowel sound—and a consonant sound of its own. This last sound is generally heard before u, and is frequently written before the other vowels. It is determined to be a consonant sound, as in the case of w, by the fact that the organs of articulation and of sound act in succession and not simultaneously.

A chapter on *Digestion* concludes the first volume of Dr. Dunglison's work on Physiology. In this part of the work, he advances an opinion, which, we think, will settle the dispute respecting absorption, that has so long distracted the Medical Profession. This is, that the veins absorb all substances that are again to pass out of the system—the lymphatics are assimilating organs, and take up nothing, but what is to be applied to the wants of the system. Experiment and analogy both appear to us to favor this opinion, and we think it probable that the sentiments of the physicians will very soon settle down upon this point.

We shall here leave the author for the present. We are unwilling to do so, however, without cordially recommending his work to the profession in this country. We know of no writer upon this subject, whose reading is more extensive or accurate in every collateral science, than Dr. Dunglison's. He has searched every department of science for the information necessary to unravel the secret operations of the animal œconomy—he has judiciously selected from the great mass of material before him, such as are best adapted to the subject—he has divested them, in a very great degree of useless speculation, and has expressed them with a degree of clearness, accuracy, and precision, which will render them intelligible to every reader. His style is, on the whole, a good one, though rather unequal. Its general character is clearness and precision, though in some parts of the work, it bears the marks of hasty composition, and is deficient in that conciseness of expression which is essential to a standard work in any profession. F.

